# Examination of the role of necroptotic damage-associated molecular patterns in tissue fibrosis

**DOI:** 10.3389/fimmu.2022.886374

**Published:** 2022-08-30

**Authors:** Xu Liu, Feng Lu, Xihang Chen

**Affiliations:** Department of Plastic and Cosmetic Surgery, Nanfang Hospital, Southern Medical University, Guangzhou, China

**Keywords:** necroptosis, RIPK3, inflammation, DAMPs, fibrosis

## Abstract

Fibrosis is defined as the abnormal and excessive deposition of extracellular matrix (ECM) components, which leads to tissue or organ dysfunction and failure. However, the pathological mechanisms underlying fibrosis remain unclear. The inflammatory response induced by tissue injury is closely associated with tissue fibrosis. Recently, an increasing number of studies have linked necroptosis to inflammation and fibrosis. Necroptosis is a type of preprogrammed death caused by death receptors, interferons, Toll-like receptors, intracellular RNA and DNA sensors, and other mediators. These activate receptor-interacting protein kinase (RIPK) 1, which recruits and phosphorylates RIPK3. RIPK3 then phosphorylates a mixed lineage kinase domain-like protein and causes its oligomerization, leading to rapid plasma membrane permeabilization, the release of cellular contents, and exposure of damage-associated molecular patterns (DAMPs). DAMPs, as inflammatory mediators, are involved in the loss of balance between extensive inflammation and tissue regeneration, leading to remodeling, the hallmark of fibrosis. In this review, we discuss the role of necroptotic DAMPs in tissue fibrosis and highlight the inflammatory responses induced by DAMPs in tissue ECM remodeling. By summarizing the existing literature on this topic, we underscore the gaps in the current research, providing a framework for future investigations into the relationship among necroptosis, DAMPs, and fibrosis, as well as a reference for later transformation into clinical treatment.

## 1 Introduction

Fibrosis is typically defined as the excessive deposition of extracellular matrix (ECM) components (such as collagen, glycoproteins, and proteoglycans), which may lead to scar formation, tissue dysfunction, and death (1). Recently, fibrosis has been suggested to be a result of tissue repair, which occurs in numerous tissues and organs, including the liver, lungs, kidneys, and fat tissues. In addition to fibroblasts, innate immune cells are also key regulators of tissue fibrosis, playing important roles in the initiation, maintenance, and resolution of tissue injury-induced inflammatory responses. The inflammatory response is thought to contribute to tissue repair and regeneration. However, it may also lead to pathological fibrosis if it becomes disorganized and chronic (2).

Necroptosis, a form of programmed necrosis, leads to rapid plasma membrane permeabilization, the release of cell contents, and exposure to damage-associated molecular patterns (DAMPs). Necroptosis has recently emerged as an important event that modulates tissue fibrosis progression. For example, hepatocyte or macrophage necroptosis is a potential contributor to chronic inflammation and fibrosis in the liver (3). Inhibition of cell necroptosis has also been reported to reduce inflammation associated with necroptosis in tissue sclerosis (4). Thus, to help understand the role of necroptosis in tissue fibrosis, we here discuss recent findings on the involvement of cell necroptosis in the regulation of tissue fibrosis development.

### 1.1 Molecular mechanisms underlying necroptosis

Necroptosis is a programmed cell death first discovered in 2005 (5). It can be activated by members of the tumor necrosis factor (TNF) family (through TNF receptor (TNFR) 1, TNFR2, TRAILR1, and TRAILR2), Fas ligand, toll-like receptors (TLR), lipopolysaccharides (LPS), double-stranded RNA (dsRNA), and genotoxic stress. Furthermore, different physical-chemical stress stimuli can also initiate necroptosis, including ATP-depletion, radiation, ischemia-reperfusion injury, glutamate, and lipid overload (5, 6).

**Figure 1:**
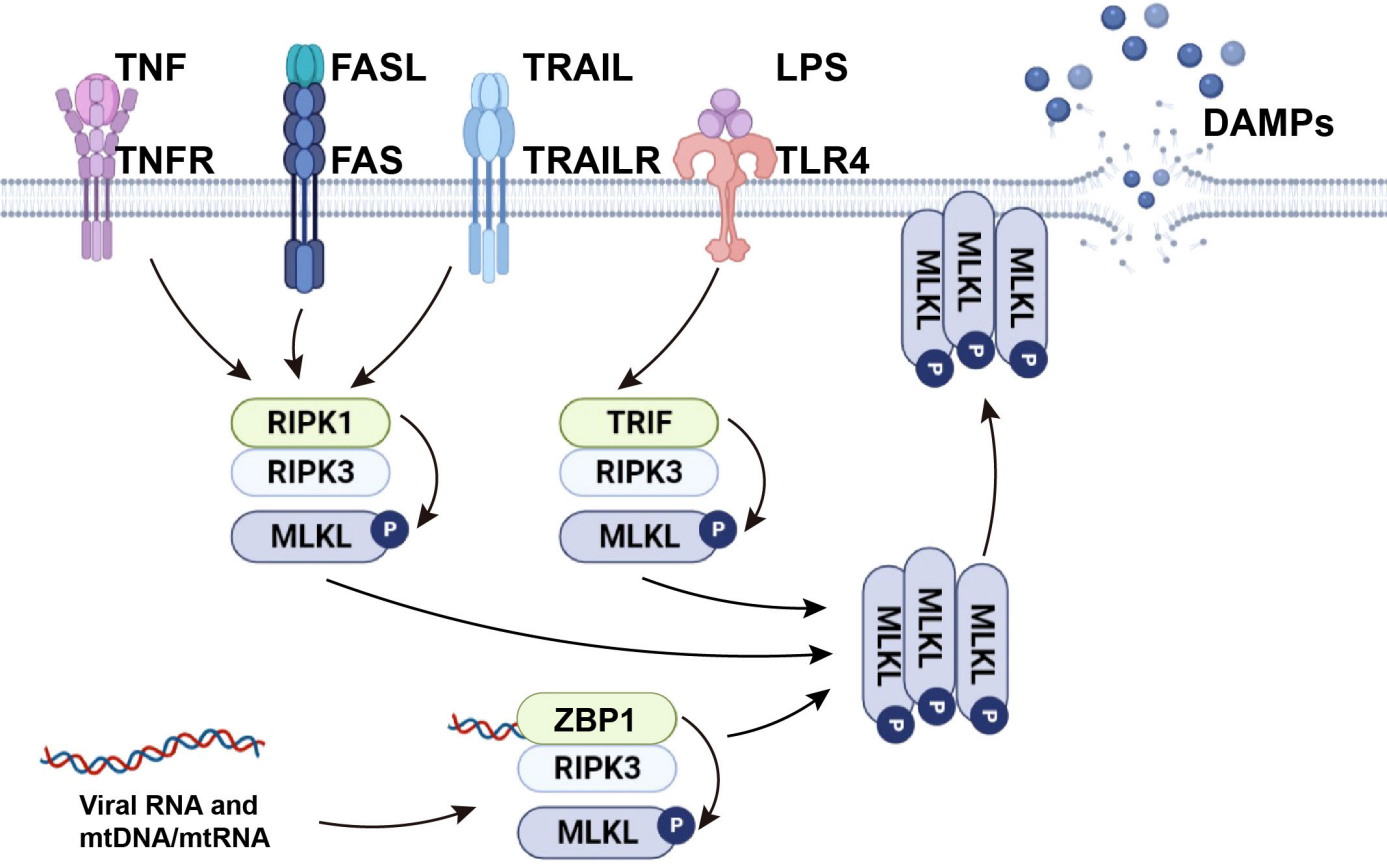
The mechanism of necroptosis. TNF and death ligands, including FasL and TRAIL, initiate necroptosis by inducing the formation of necrosome complexes. LPS activates necroptosis by TRIF-mediated necrosome complex formation. Viral RNA and cellular mtDNA/mtRNA bound to ZBP1 cause RIPK1-independent necroptosis through the ZBP1-RIPK3 complex. Activated RIPK3 phosphorylates MLKL and causes MLKL oligomerization. The oligomerized MLKL migrates to the plasma membrane, where it induces necroptosis by initiating membrane rupture and releasing DAMPs.

The necroptosis signaling pathway is primarily modulated by activation of receptor-interacting protein kinase (RIPK) 3, which phosphorylates mixed-lineage kinase domain-like protein (MLKL), mediating MLKL oligomerization. In the absence of caspase-8, RIPK1 recruits and phosphorylates RIPK3 to form the ripoptosome and then phosphorylates MLKL to form the necrosome (a complex containing RIPK1, RIPK3, and MLKL). After RIPK3 phosphorylates MLKL, p-MLKL undergoes conformational changes and oligomerization and is transferred to phosphatidylinositol-rich patches on the plasma membrane to form macropores. This can lead to necroptotic cell death by allowing ion influx, cell swelling, and membrane rupture, followed by the uncontrolled release of intracellular material, mainly DAMPs (7, 8). In addition to RIPK1-independent activation, necroptosis can be also triggered by activation of TLR3 and TLR4 by dsRNA and LPS respectively, through TIR-domain-containing adaptor-inducing interferon-β (TRIF)-dependent activation of RIPK3. Viral RNA and the DNA/RNA released from damaged mitochondria (mtDNA/mtRNA) can induce necroptosis by Z-DNA binding protein 1 (ZBP1)-dependent activation of RIPK3. Activated RIPK3 then phosphorylates MLKL (9). From this, it can be concluded that RIPK3 and MLKL are integral components of necroptosis regardless of their upstream trigger (**Figure 1**).

### 1.2 Release of necroptotic DAMPs

DAMPs secreted from necroptotic cells have been identified as potent activators of the immune system that trigger fibrosis. Necroptotic DAMPs can be divided into two categories (1): molecules that perform noninflammatory functions in living cells (such as HMGB1) and acquire immunomodulatory properties when released, secreted, modified, or exposed on the cell surface during cellular stress, damage, or injury, or (2) alarmins, molecules that possess cytokine-like functions (such as IL-1α, S100A8, and IL-33) that can be stored in cells and released upon cell lysis, whereupon they contribute to the inflammatory response (10).

Necroptotic DAMPs can directly activate profibrotic responses of nonimmune cells, such as epithelial cells, endothelial cells, and fibroblasts. For example, necroptotic macrophages are reported to promote collagen synthesis in fibroblasts *via* the release of IL-6 and TNF-α during fat graft fibrosis (11). Experiments have also shown that self-DNA produced by necroptotic glomeruli and tubules in the kidney activates cyclic guanosine monophosphate-adenosine monophosphate synthase and is absent in melanoma 2, thereby inducing fibroblast proliferation and migration (12, 13).

In addition, the innate immune response is proposed to contribute to the pathogenesis of fibrosis (2). Necroptotic DAMPs, including ATP and HMGB1, can activate innate immune cells such as neutrophils and macrophages. Activation of these cells leads to the production of various cytokines and chemokines, which in turn recruit inflammatory cells and promote collagen secretion in fibroblast or hepatic stellate cells (HSCs) (14). In addition, group 2 innate lymphoid cells can produce more IL-13 when exposed to IL-33, which leads to the activation of fibroblasts or HSCs.

### 1.3 Necroptotic DAMP-sensing receptors and inflammasomes in tissue fibrosis

**Figure 2:**
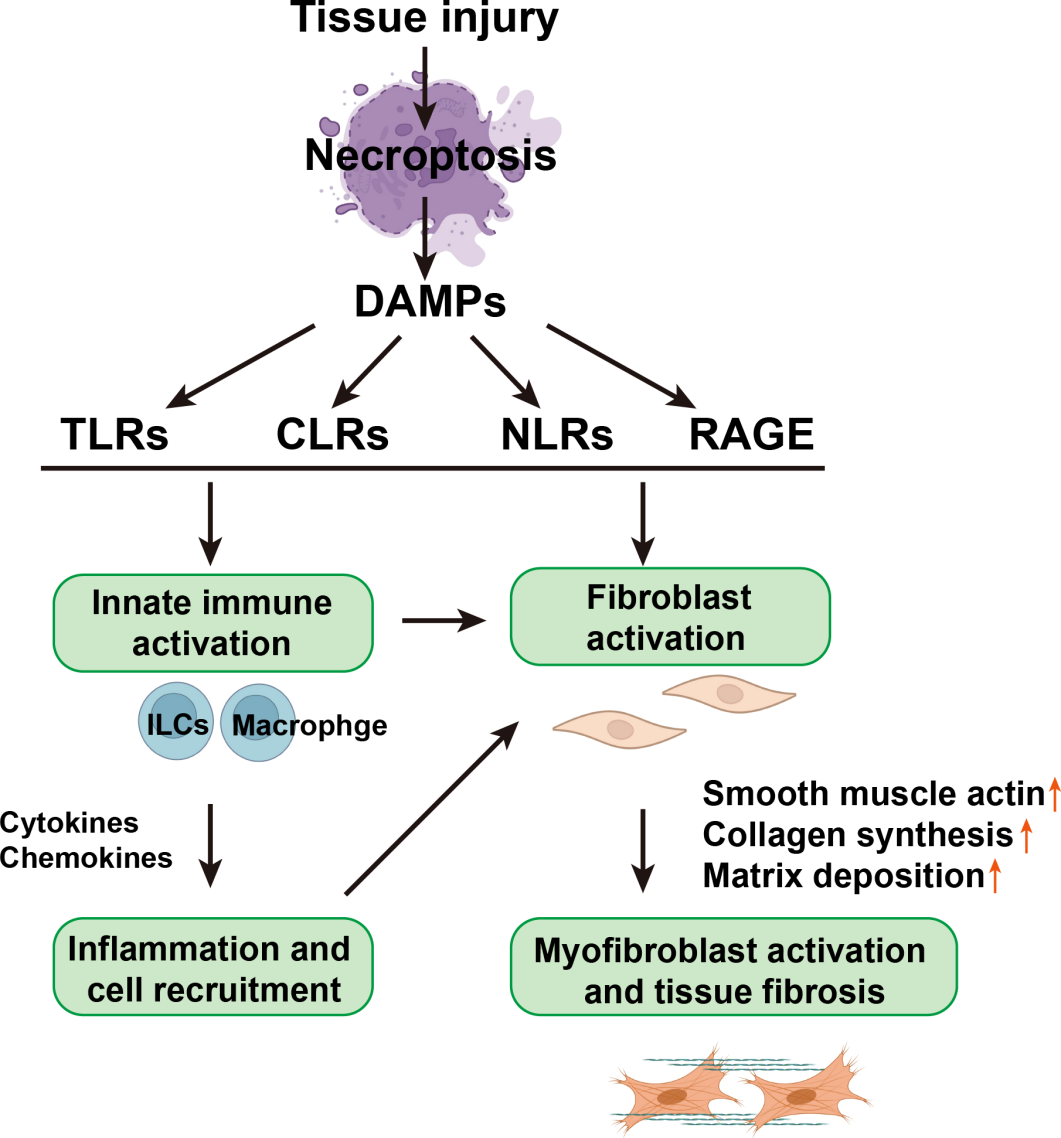
The relationship between necroptotic DAMPs, inflammation, and fibrosis. Necroptotic DAMPs can initiate immune responses and profibrotic responses of nonimmune cells, such as epithelial cells, endothelial cells, and fibroblasts, through the activation of PRRs, which include TLRs, CLRs, and NLRs. Necroptotic DAMPs can also be sensed by non-PRR DAMP receptors, such as RAGE. On the one hand, DAMPs can directly activate fibroblasts and then activate myofibroblasts, directly causing tissue fibrosis; on the other hand, DAMPs stimulate innate immune cells, such as innate lymphoid cells (ILCs) and macrophages, to secrete cytokines and chemical factors, thereby triggering cell recruitment and inflammation and further activating fibroblasts.

The recognition of DAMPs relies on the cell surface, endosomal, and cytosolic pattern recognition receptors (PRRs) that include TLRs, NOD-like receptors (NLRs), C-type lectin receptors (CLRs), and receptors for advanced glycation end products (RAGE). These DAMP-sensing receptors, together with inflammasomes, might represent an important pathway responsible for converting self-limited regenerative repair into an unresolved fibrotic process (15) (**Figure 2**).

Necroptotic DAMP receptors have also been reported to promote fibrosis. For example, in ischemia-reperfusion injury animal models, gene knockout or inhibition of TLR can alleviate systemic inflammation and tissue fibrosis in the liver, kidney, and intestine (16). Macrophage-inducible C-type lectin is a CLR that mediates collagen secretion in macrophages during adipose tissue fibrosis (17). Moreover, NLRs and other PPPs exhibit significant interplay. Some NLRs can detect DAMPs and stimulate the expression of proinflammatory genes, while other NLRs require the participation of other PRRs in order to promote the processing and secretion of the key proinflammatory cytokines, IL-1β and IL-18, which are involved in the pathogenesis of various inflammatory diseases (including systemic sclerosis (SSc), atherosclerosis, and arthritis), leading to tissue fibrosis (18).

Inflammasomes are the bridge signaling between DAMP identification and the immune response. According to the differently activated caspases, inflammasomes are divided into two categories: classical and non-classical. Classical inflammasomes mainly activate caspase-1, including NOD-like receptor protein 1 (NLRP1), NOD-like receptor protein 3 (NLRP3), NOD-like receptor C4 (NLRC4), and NLRC5; nonclassical inflammasomes mainly activate caspases other than caspase-1, including AIM2, etc. (19). The NLRP3 inflammasome, the most studied, is typically composed of NLRP3, ASC, and procaspase-1. The inflammasome complexes induce cells to produce cytokines. Cumulative evidence shows that inflammasomes are involved in the formation of fibrosis in multiple organs (20–22). Thus, the next specific introductions will be made according to different receptors and their corresponding inflammasomes.

Next, specific introductions will be made according to different receptors and their corresponding inflammasomes:

#### 1.3.1 TLRs

So far, 10 TLRs have been found in humans and 12 in mice, including human TLR1-TLR10, mouse TLR1-TLR9, and TLR11-TLR13. When combined with specific ligands, the TLR signaling pathways are activated, leading to the induction of inflammatory cytokines and/or type I interferon (23). With the exception of TLR3, all TLRs communicate *via* the MyD88 to trigger the production of proinflammatory cytokines by activating nuclear factor (NF)-B and mitogen-activated protein kinases. The generation of proinflammatory cytokines and profibrotic mediators, which aid in the promotion of tissue fibrosis, is caused by the recognition of DAMPs by TLRs in damaged or necroptotic cells.

Due to tissue specificity, different DAMPs are released from distinct tissue when they undergo necroptosis. In skin-related fibrosis diseases, HMGB1 binds to TLR2 and TLR4 to stimulate the release of TNF, IL-1, IL-6, and IL-8, which increases the expression of α-SMA and collagen I in epidermal cells, keratinocytes, and dermal fibroblasts, thus forming hypertrophic scar (24, 25). Fibronectin-EDA and tenascin-C combine with TLR4 to increase the expression of TGF-β, TIMP-1, and collagens in myofibroblasts and plasmacytoid dendritic cells, thus inducing epidermal fibrosis and sclerosis. TLR8 is also involved in systemic sclerosis by binding to tenascin-C (26, 27). According to studies, the detection of DAMPs and TLRs stimulates the development of microRNA-155, which in turn boosts the expression of the NLRP3 inflammasome and causes SSc to experience persistent chronic inflammation and ECM secretion (28, 29). Fibronectin-EDA and TLR2/4 were matched in cardiac fibrosis, causing myofibroblasts to begin producing collagen I and III (30). Following the combination of S100A8/A9 and TLR4, which was intended to produce cardiac fibrosis, the expression of α-SMA, collagen 1A1, and 3A1 mRNA was also enhanced in fibroblasts (31, 32).In pulmonary fibrosis, HSP90 and uric acid react with TLR2/4, aggravating the expression of TIMP-1 and the synthesis of fibronectin (33). S100A4 is also associated with TLR4 leading to elevated α-SMA expression and increased collagen I synthesis during pulmonary fibrosis (34). Mitochondrial DNA unites with TLR9 on alveolar epithelial cells to trigger the expression of TGF and IL-1/6, which leads to increased α-SMA and collagen I synthesis during pulmonary fibrosis (35). During renal fibrosis, HMGB1 and fibrinogen bind to TLR2 on tubular epithelial cells and interstitial cells, leading to enhanced synthesis of proinflammatory cytokines, and increased number of myofibroblasts, causing raised in α-SMA expression and collagen synthesis (36, 37).

#### 1.3.2 NLRs

A total of 22 receptors of the NLR family have been found in humans. These receptors are positioned in the cytoplasm of the cells (38). Depending on the structure, human NLRs have been divided into four subgroups (39). Among them, the NLR-pyrin domain containing 3 (NLRP3) is restricted with DAMPs.

DAMPs that are recognizable by NLRP3 mainly include ATP, hyaluronic acid, sodium urate, uric acid, and cholesterol crystals (15, 40). In lung-associated fibrosis, ATP and uric acid are recognized by NLRP3, causing the recruitment of inflammatory cells IL-1 and TIMP1, worsening inflammation and promoting extracellular collagen synthesis, leading to pulmonary fibrosis (41). A similar situation also occurs in mitochondrial DNA-related pulmonary fibrosis. By binding to NLRP3 and activating NLRP3 inflammasomes, the expressions of TGF-β, IL-1β, IL-6, and TNF-β are increased, stimulating collagen I and α-SMA synthesis (35). S100A9, released from necroptosis in the liver, binds to NLRP3 on HSCs and myofibroblasts, triggering an inflammatory response and generating extracellular collagen synthesis, resulting in liver fibrosis (42). Increased TNF and IL-17 expression, which is mediated by the NLRP3 inflammasome, is a major contributor to liver fibrosis and inflammation (43). In animal models of NAFLD, inhibition of NLRP3 may considerably improve the prognosis and liver fibrosis. By activating Kupffer cells, DAMPs cause the production of NLRP3, pro-caspase-1, and pro-IL-1 components of the inflammasome-related pathway (44). TLRs and NF-kB pathways allow DAMPs like HMGB1 to activate the NLRP3 inflammasome (45). By phosphorylating Smad2/3, the NLRP3 inflammasome causes the expression of α-SMA in HSCs and the conversion of hepatocytes to EMT, which produces collagen type 1 and results in liver fibrosis (46). Additionally, studies have shown that the NLRP3 inflammasome is directly implicated in the development of liver fibrosis and may be directly expressed and activated in HSCs without the need for cytokines (47). In cardiac fibrosis, NLRP3 also activates Smad2/3 to promote IL-1 expression and the transformation of CFs into MFs (48, 49). IL-36 signaling contributes to tubulointerstitial lesions in kidney-related inflammation and fibrosis *via* activating the NLRP3 inflammasome and the IL-23/IL-17 axis (50). Additionally, NLRP3 activates Smad2/3 to phosphorylate vascular endothelial cells into myofibroblasts, which increases the production of α-SMA and matrix metalloprotein 9 (MMP9) (51).

Silencing NLRC5 reduced cardiac fibrosis by blocking the TGF-1/Smad3 signaling pathway, which is a critical regulator of cardiac fibrosis (52). Additionally, NLRC5 causes ECs to phosphorylate Smad2/3 and activate EMT, which results in cardiac fibrosis (53). NLRC5 could thus be a diagnostic and therapeutic target for heart fibrosis in diabetic cardiomyopathy. Maternally expressed gene 3 (MEG3), a long non-coding RNA (lncRNA), may target and control NLRC5, prevent the activation of HSCs through the TGF-1/Smad and NF-kB signaling pathways, and reverse liver fibrosis (54). By controlling the TGF-1/Smad signaling pathway in chronic kidney disease, NLRC5 may decrease renal fibroblast activation and improve renal fibrosis (55, 56).

NLRC4 may create an IL-1R antagonist (IL-1α) *via* NF-kB, which binds to IL-1α and inhibits the development of fibrosis, unlike other inflammasomes that promote inflammation and fibrosis (57). Furthermore, it has been shown that NLRC4 works to prevent liver fibrosis, encourage hepatocyte regeneration, and reverse it (21).

AIM2, which is triggered by the presence of dsDNA in DAMPs, is a representative of the nonclassical inflammasome. In IPF, AIM2 activation causes the noncanonical inflammasome (caspase-4-dependent) to become active. This raises the production of IL-1α, which in turn enhances the release of TGF-β, causing fibrosis (22). The pathophysiology of pulmonary fibrosis involves mitochondrial oxidation-driven overactivation of the AIM2 inflammasome *via* the production of IL-1α, IFN-α, and TGF-β. Blocking the AIM2 inflammasome aids in reducing lung fibrosis and inflammation brought on by radiation (58, 59).

#### 1.3.3 CLRs

CLRs can be classified into four groups with different cytoplasmic signaling motifs (60). One of the CLRs that is closely associated with DAMPs (SAP130, cholesterol sulfate, and cholesterol crystal) is MINCLE (61). MINCLE evokes the production of several cytokines and chemokines, including TNF-α, IL-6, MIP-2, and CXCL1 (62–64). In IPF, as a novel biomarker, SAP130 binds to MINCLE to increase the secretion of interstitial inflammatory factors in the lung and eventually cause interstitial fibrosis (65). In the liver, MINCLE was mainly found in Kupffer cells and macrophages. When DAMPs make contact with the MINCLE on the cell membranes, it stimulates the cells to secrete inflammatory factors and promotes M1 macrophage infiltration and collagen synthesis, eventually contributing to liver fibrosis (66). DAMPs generated by dying adipocytes, which are important in the aggregation of macrophages to create crown-like structures, promote MINCLE in macrophages during the development of obesity. Myofibroblasts were created as a result of MINCLE activation, which also caused the production of fibrosis-related genes (17, 67). Inhibition of renal fibrosis by suppressing MINCLE-induced inflammation can alleviate the progression of chronic kidney disease (68). By regulating the expression of macrophage colony-stimulating factor (M-CSF) to control fibrosis, C-type lectin-like receptors (CTLRs) play a significant role in interstitial lung disease (69).

#### 1.3.4 Other receptors

In addition to the aforementioned PRRs, other non-PRR DAMP receptors are also involved in fibrosis induced by necroptotic DAMPs. RAGE is a member of the immunoglobulin superfamily of receptors, which include the alveolar epithelial type I cells of the lung (70). HMGB1, S100 family, and others could bind to RAGE. For example, when combined with RAGE, HMGB1 and S100A8/A9 are suggested to play a role in pulmonary fibrosis (71). By binding to RAGE, it stimulates the infiltration of alveolar macrophages, causing fibroblasts to differentiate into myofibroblasts; subsequently, bronchiolar epithelial cells and alveolar epithelial cells secrete inflammatory cytokines, resulting in extracellular collagen deposition and ultimately pulmonary fibrosis (72, 73). Similar findings were also demonstrated in liver fibrosis and renal fibrosis (74, 75). The roles of the remaining receptors and their corresponding DAMPs in tissue fibrosis are shown in **Table 1**.

**Table 1:** Functions of necroptotic DAMPs and their receptors in fibrosis.

Organ/Tissue	Disease	DAMPs	Receptor	Related Cells	Function
Skin	Hypertrophic scar	HMGB1	RAGE TLR2 TLR4	Dermal fibroblast Keratinocyte Epidermal cell	α-Smooth muscle actin promoter↑ Collagen I↑ (24, 25)
	Scar	S100A8/A9		dermal keratinocyte Fibroblast	Epithelial-mesenchymal transition↑ Dermal keratinocytes↑ Fibroblast↑ (76, 77)
	Chronic cutaneous fibrosis	Fibronectin-EDA	TLR4	Myofibroblast	Collagen↑ Myofibroblast↑ (26)
	Systemic sclerosis	Tenascin-C	TLR4 TLR8	Myofibroblast Plasmacytoid dendritic cells	TGF- β↑ TIMP-1↑ Fibronectin↑ (27)
	Systemic sclerosis	ATP	P2X P2Y	Fibroblast	Collagen I↑ IL-6↑ (78)
Liver	Liver fibrosis	HMGB1	RAGE	HSC Hepatocyte Kupffer cell	Collagen I↑ (74, 79)
	Liver fibrosis	IL-33	ST2	HSC Endothelial cell	Proinflammatory cytokines↑ Collagen I↑ (80)
	Chronic hepatic diseases	ATP	P2Y2 P2X4	HSC Hepatocyte Hepatic myofibroblast	Collagen I↑ Inflammasome↑ (81)
	Nonalcoholic steatohepatitis (NASH)	Mitochondrial DNA		HSC	Collagen↑ α-SMA↑ TIMP-1↑ (82)
	Liver fibrosis	S100 family	TLR4 RAGE NLRP3	HSC Myofibroblast	Cytokines↑ Chemokines↑ Collagen↑ (42, 83)
Heart	Myocardial fibrosis	HMGB1	TLRs RAGE	Cardiomyocyte Cardiac fibroblast	Collagen I↑ Collagen III↑ TGF-β1↑ Fibroblast↑ (32, 84)
	Cardiac fibrosis	S100A8/A9	TLR4	Fibroblast Myofibroblast	Fibroblast growth factor 2↑ Proinflammatory cytokines↑ Fibroblast↑ (31, 32)
	Hypertensive cardiac fibrosis	Tenascin-C		Myocardial cell Fibroblast	Proinflammatory/profibrotic cytokines↑ Collagen I/III↑ (85)
	Myocardial Infarction	Fibronectin-EDA	TLR2 TLR4	Cardiac fibroblast Myofibroblast	Cytokines and chemokines↑ Myofibroblast↑ Collagen I/III↑ (30)
Lung	Pulmonary fibrosis	HMGB1	RAGE TLR2 TLR4	Alveolar macrophage Bronchiolar epithelial cell Alveolar epithelial cell Fibroblast	Fibroblast↑ Collagen↑ (72, 86)
	IPF	SAP130	MINCLE	Alveolar epithelial cell	Inflammatory cytokines↑ (65)
	Pulmonary fibrosis	S100A8/A9	RAGE	Fibroblast	Fibroblasts’ differentiation to myofibroblasts↑ Inflammatory cytokines↑ Collagen↑ (73)
	Pulmonary fibrosis	S100A4	TLR4 RAGE	fibroblast	α-SMA↑ Collagen I↑ (34)
	Pulmonary fibrosis	HSP 90	TLR2 TLR4		Collagen I↑ Fibronectin↑ (33)
	Pulmonary fibrosis	Uric acid	NLRP3 TLR2 TLR4		Collagen↑ Inflammation↑ TIMP-1↑ (87)
	Pulmonary fibrosis	Tenascin-C		Fibroblast Myofibroblast	Collagen↑ TGF-β↑ α-SMA↑ (88)
	Pulmonary fibrosis	Mitochondrial DNA	TLR9 NLRP3	Alveolar epithelial cell Myofibroblast Fibroblast	TGF-β↑ Collagen I↑ α-SMA↑ IL-1β↑ IL-6↑ TNF-β↑ (35)
	pulmonary fibrosis	ATP	NLRP3 P2X7	Pulmonary epithelial cell Endothelial cell	TIMP-1↑ IL-1β↑ Collagen↑ (41)
Kidney	Renal fibrosis	HMGB1	TLR2	Tubular epithelial cell Interstitial macrophage M1 phenotype Tubulointerstitial cell	Proinflammatory cytokines↑ M1 macrophage transition↑ Myofibroblast↑ Interstitial myofibroblast↑ Collagen↑ (36, 89)
	Renal damage and fibrosis	S100A8/A9	RAGE TLR4	Granulocyte Tubular epithelial cell Myofibroblast	Epithelial–mesenchymal transition↑ α-SMA↑ Collagen↑ (75)
	Renal fibrosis	IL-33	ST2	Interstitial cell Tubular epithelial cell	Vimentin↑ α-SMA↑ Fibroblast↑ Myofibroblast↑ Proinflammatory cytokine and chemokine↑ (90)
	Renal fibrosis	Fibrinogen	TLR2 TLR4	Interstitial cell Fibroblast	Collagen↑ α-SMA↑ (91)

↑ represents the increased expression of corresponding inflammatory factors, cytokines, and extracellular matrix(a-SMA and Collagen I/III) in tissues and cells.

## 2 Necroptotic DAMPs in different types of tissue fibrosis

### 2.1 Wound healing

The wound-healing process is divided into three stages: inflammation, regeneration, and remodeling. Keratinocytes and dermal fibroblasts are thought to be key cells in wound re-epithelialization and closure. Recently, necroptotic cell death has been observed at the site of chronic wounds (92). Khandelwal et al. reported that protecting keratinocytes from necroptosis using a surfactant polymer wound dressing can favor wound healing (25). Another study showed that RIPK3 deficiency fully prevents skin lesion development in animals with epidermal keratinocyte-restricted deficiency of the adaptor protein FAS‐associated death domain by inhibiting deubiquitinase enzyme cylindromatosis and TNFR1 signaling (92). In addition, FAS-associated death domain or caspase-8 deficiency causes RIPK3-dependent keratinocyte necroptosis and induces the release of IL-1α, which worsens skin inflammation (93). HMGB1, an ubiquitous nuclear protein, is present in almost all cell types in the nucleus and cytoplasm. The release of HMGB1 from necroptotic keratinocytes has been observed during chronic wound healing (25). The inflammatory function of HMGB1 is mediated by its binding to cell-specific receptors such as RAGE, TLR2, TLR4, and TLR9. Serum HMGB1 levels are higher in patients with scleroderma and are correlated with skin fibrosis (94). Reactive oxygen species–induced dermal fibroblast necroptosis has also been observed in skin wound healing in diabetes. Sirtuin 3 activation has been suggested as a potentially promising therapeutic strategy for skin wound healing in diabetes through the prevention of fibroblast necroptosis (95). According to these findings, necroptosis exerts a damaging effect on wound healing. In addition, chronic wound healing may be accelerated by the expression of essential regulatory elements in necroptosis.

### 2.2 Liver fibrosis

Liver fibrosis is closely associated with chronic liver disease. Cell death in chronic liver disease has been reported to contribute to chronic hepatocyte turnover, recruitment of immune cells, activation of hepatic stellate cells, and, thereby, the development of liver fibrosis (96).

In steatohepatitis (NASH), RIPK3-mediated necroptosis has been shown to be upregulated in both human and dietary-related mouse NASH models. Furthermore, studies have revealed that inhibition of necroptosis *via* RIPK3 ablation in dietary-related mouse models reduces NASH-related liver inflammation and fibrosis (97, 98). RIPK3 mediates liver injury, inflammation, induction of hepatic progenitor cells/activated cholangiocytes, and liver fibrosis through a pathway suppressed by caspase-8 (99). In addition, in patients with NASH, serum concentrations of RIPK1 and MLKL increase with activity, and RIPK1 inhibition improves NASH features in high-fat diet-fed mice and reverses steatosis *via* an MLKL-dependent mechanism (100). Mohammed et al. also revealed that increased necroptosis in the liver plays a role in the increased inflammation and fibrosis observed in NASH mice. They found that markers of pro-inflammatory M1 macrophages, NLRP3 inflammasomes, transcript levels of pro-inflammatory cytokines and chemokines such as TNF-α, IL-6, IL-1β, and Ccl2, expression of antioxidant enzymes and heat shock proteins, and markers of fibrosis are significantly increased in NASH mice; however, treatment of NASH mice with a necroptosis inhibitor reverses these conditions (101). In addition, hepatocyte injury or death has been reported to release mitochondrial DAMPs. Mitochondrial DNA, the main active component of mitochondrial DAMPs, can trigger the activation of fibrosis *in vivo* and *in vitro*, leading to reduced liver fibrosis. Mitochondrial DNA levels have been found to be significantly elevated in the serum of patients with fibrotic NASH (82).

Significantly higher serum levels of IL-33 have been observed in patients with liver cirrhosis compared to control. A previous study reported that hepatic expression of IL-33 is both required and sufficient for severe hepatic fibrosis *in vivo*. IL-33 does not mediate fibrosis through direct effects on hepatic stellate cells but rather through IL-33-induced increases in IL-13 production in group 2 innate lymphoid cells and subsequent hepatic stellate cell activation (102). The mechanism of IL-33 release during liver cirrhosis has not been elucidated; however, necroptosis has recently been shown to directly induce the release of nuclear IL-33 in its full-length form (80). Therefore, we propose that IL-33 may be considered a necroptotic DAMP that contributes to liver cirrhosis-related fibrosis.

During age-related liver fibrosis, it is worth noting that necroptosis contributes to an increase in the number of proinflammatory M1 macrophages and expression of proinflammatory cytokines (TNF-α, IL-6, and IL-1β), as well as total collagen content in aging livers. Inhibition of necroptosis by necrostatin-1 (Nec-1) has been shown to reduce elevated collagen deposition in old mice by 2.5-fold to levels similar to those in young mice (3).

Liver RIPK3 and HMGB1 protein expression are correlated with fibrosis stage in patients with chronic hepatitis C virus infection, primary biliary cirrhosis, or alcoholic steatohepatitis (74). Recently, increasing evidence has suggested a role for HMGB1 derived from necroptotic hepatocytes in promoting liver fibrosis (103) and inhibiting the necroptotic pathway-attenuated HMGB1 cytoplasmic translocation and liver damage (104). HMGB1 has been reported to stimulate hepatic stellate cell migration and upregulate type I collagen expression (74). Furthermore, HMGB1 plays an important role in recruiting neutrophils to the injured site of necrotic tissue, resulting in aseptic inflammation, injury amplification, and a reduced survival rate. The S100-calcium-binding protein family, which contains critical DAMP molecules, has also been linked to liver necroinflammation in chronic hepatitis (83). Moreover, necroptosis- and S100A9-dependent NLRP3 inflammasome activation has been reported to induce liver necroinflammation (42). Upregulation of S100A11, a member of the S100 family, significantly contributes to inflammation and fibrosis development in hepatic tissues, which are key drivers of hepatocarcinogenesis (105). In addition, ECM-DAMPs (versican (106), thrombospondin-1 (107), periostin (108), and laminin (109)) promote the local inflammatory immune response and chemokine immune cell recruitment and inflammation, thereby stimulating liver fibrosis (110).

In summary, numerous studies have illustrated that necroptosis produces DAMPs and causes inflammation, eventually leading to fibrosis. Thus, the formation of the necroptosis-DAMP-inflammation axis plays an important role in liver fibrosis.

### 2.3 Heart fibrosis

Aggregation of the ECM protein network is a feature of myocardial fibrosis (111). Myocardial collagen formation is stimulated by oxidative stress and inflammation, leading to fibrosis (112, 113). Myocardial remodeling and fibrosis are caused by several cardiac disorders. Previous studies have revealed that necroptosis is involved in a variety of cardiac disorders. For example, coronary atherosclerosis is one of the leading causes of coronary heart disease (114). Myocardial vascular stenosis and occlusion result from severe atherosclerosis, leading to myocardial ischemia (MI), necrosis, and cardiac remodeling. According to previous studies, expression of RIPK3 and MLKL mRNA increases in atherosclerotic plaques (115), whereas RIPK3 ^KO^ mice with atherosclerosis exhibit reduced inflammation followed by death (116). In an MI mouse model, RIPK3-dependent necroptosis governs post-ischemic unfavorable remodeling (117). A previous study found that cardiac expression of RIPK3 in MI is upregulated, and RIPK3-deficient animals have a considerably higher ejection fraction and reduced hypertrophy. Ischemia and oxidative stress induce necroptosis, apoptosis, and inflammation in myocardial cells, which leads to myocardial remodeling and heart failure (118).

Myocardial ischemia-reperfusion injury is closely related to necroptosis (118). It has been demonstrated that the RIPK1 inhibitor Nec-1 protects the heart from ischemia-reperfusion injury (119). Myocardial infarction and other ischemic heart diseases lead to cell death, releasing DAMPs such as HMGB1, double-stranded DNA, and ATP (32), activating TLRs and inducing the expression of pro-inflammatory chemokines and cytokines, which result in changes to the ECM (types I and III collagen), fibrosis, and systolic dysfunction (120).

In conclusion, these studies demonstrate that necroptosis plays a significant role in a variety of cardiac disorders. It can limit or alleviate cardiac inflammation and oxidative stress, consequently decreasing cardiomyopathy, excessive ECM synthesis, and fibrosis by blocking major pathway regulatory elements.

### 2.4 Lung fibrosis

Idiopathic pulmonary fibrosis (IPF) is the most prevalent and severe idiopathic non-severe interstitial illness of unknown cause. In IPF, alveolar epithelial cell (AEC) damage and repair failure are followed by fibroblast/myofibroblast activation, fibrosis, and scar formation in the lung parenchyma, leading to lung function loss and respiratory failure (121). RIPK3-mediated necroptosis in AECs contributes to the development of pulmonary fibrosis by DAMP generation, which is part of the IPF etiology (122). RIPK3 expression is higher in IPF lung tissue, particularly in AECs. Bleomycin enhances HMGB1 and IL-1 levels in mouse lung tissue and elevates RIPK3 expression in AECs. Bleomycin-induced lung inflammation and fibrosis have been successfully reduced in RIPK3^KO^ mice. RIPK3 expression is elevated in the lungs of patients with chronic obstructive pulmonary disease. Nec-1 can reduce inflammation in mice exposed to cigarette smoke (123). Inflammatory cytokines, such as extracellular ATP and DAMPs, have been found to be elevated in the airways of patients with chronic obstructive pulmonary disease (124). Asthma is a common respiratory condition characterized by airway hyperresponsiveness, inflammation, and fluctuating airflow restriction. Eosinophil disintegration and particle release have been linked to airway inflammation. As decreasing RIPK3 expression reduces eosinophil disintegration, necroptosis may be involved in eosinophil death (125). However, RIPK3-deficient animals have been shown to be resistant to allergen-induced asthma (126). Necroptosis is critical for the development of acute lung damage and is indicated by an increase in HMGB1 in the bronchoalveolar lavage fluid of individuals with acute lung damage (127). The concentration of phosphorylated RIPK3 and MLKL increased as the LPS dosage increased in an acute respiratory distress syndrome animal model. Deletion of Nec-1 or RIPK3 genes inhibits necroptosis and improves the lung status of animals with acute respiratory distress syndrome (128). These findings indicate that necroptosis plays a key role in various lung diseases and that DAMPs cause inflammation, which contributes to lung fibrosis.

In the abovementioned severe pulmonary inflammatory diseases, lung epithelial cells and innate immune cells undergoing necroptosis can secrete numerous DAMPs. This triggers the release of proinflammatory cytokines and further aggravates severe inflammation, changing the steady-state lung environment into one with inflammation and amplification of DAMP release, resulting in pulmonary fibrosis. The released DAMPs then bind to TLRs. TLR4 has been shown to promote fibrosis in the lungs (86). HMGB1 may also be a potential target for treating inflammation and improving pulmonary fibrosis. HMGB1 levels are elevated in patients with IPF. The potential pathogenic role of HMGB1 in IPF has been supported by experimental animal models, indicating that anti-HMGB1 antibodies can prevent bleomycin-induced pulmonary fibrosis (72).

### 2.5 Adipose tissue fibrosis

After an injury, adipose tissue undergoes a series of different reactions, including cell necrosis, local or systemic inflammatory responses, and complete or incomplete regeneration repair caused by fibrosis or scarring. Adipose tissue fibrosis is difficult to reverse in most cases, posing significant therapeutic challenges and causing considerable discomfort to patients. Adipose tissue fibrosis, similar to other fibrotic disorders, is linked to an increase in ECM storage and synthesis (129, 130).

The most frequently observed ECM proteins in healthy and fibrotic adipose tissues are fibronectin and collagen, respectively. The amount of types I, III, V, and VI collagen is greater in obese and diabetic mice than in normal mice (131). RIPK3 is overexpressed in the white adipose tissue of obese mice fed a choline-deficient high-fat diet. Genetic inactivation of RIPK3 promotes caspase-8-dependent adipocyte apoptosis and increases white adipose tissue inflammation (98). Adipose tissue damage causes changes in adipose cell volume and mass, leading to inflammation, adipose tissue imbalance, and eventually fibrosis (132). Some experiments have also shown that a lack of TLR4 can effectively inhibit the inflammatory response and obesity in adipose tissue (133). Another cause of fat fibrosis is hypoxia. Long-term hypoxia can cause damage, fibrosis, cell aging, and the death of necrotic adipocytes (134).

With advancements in fat transplantation technology, this technique is widely used and recognized. However, due to various factors, this method also has many complications. The most common complications, no/low micrograft retention, infection, oil cysts, and calcification, are associated with graft fat necrosis (135). At present, necrosis of transplanted adipose tissue remains to be further explored, but it is worth noting that complications such as calcification and oil cysts are closely related to fibrosis. Previous studies have shown that necroptosis plays an important role in the occurrence of fibrosis. Therefore, it is reasonable to speculate that necroptosis is related to the formation of fibrosis after fat transplantation. Conversely, following injury, transplanted adipose tissue ruptures some adipocytes, causing intracellular substances to flow out of cells. These can subsequently be used as DAMPs to cause an inflammatory response and aggravate fibrosis. Injured cells in adipose tissue secrete DAMPs such as LPS and fatty acids, activate the TLR4 pathway, and induce an inflammatory response (136). Several proinflammatory chemokines, including TNF-α, inducible nitric oxide synthase, IL-6, IL-8, C-reactive protein, transforming growth factor-β1, soluble intercellular adhesion molecule-1, and monocyte chemoattractant protein-1, are produced following adipocyte death (137–139). M1 macrophages are observed in large numbers in fibrotic adipose tissue. In healthy adipose tissue, permanent macrophages comprise 10–15% of stromal cells (140). The concentration of stromal cells in obese individuals increases by 45%–60% (141, 142). Adipose stromal cells (ASCs) tend to develop into proinflammatory cells in the presence of M1 macrophages. TNF, inducible nitric oxide synthase, and IL-6 are proinflammatory cytokines released by macrophages that trigger this alteration (142, 143). Exposure of ASCs to substances released by M1 macrophages has been shown to enhance ECM remodeling. In addition, ASCs have a proinflammatory profile that causes them to proliferate and migrate while decreasing their differentiation potential (143). This implies that in the presence of the M1 macrophage secretory factor, macrophages induce the formation of profibrotic ASCs. Thus, M1 macrophages play a key role in promoting fat fibrosis. Another study discovered that apoptotic adipocytes can cause fibroblast necroptosis, which promotes collagen production *via* paracrine pathways (11). In mice, fibrosis has been found to increase following fat transplantation. Macrophages generate macrophage foam cells surrounding apoptotic fat cells or large oil droplets, engulfing oil droplets and other cell debris before necroptosis. Macrophage foam cell necroptosis causes fibrosis by altering the expression of types I and VI collagen in fibroblasts *via* a paracrine pathway that involves inflammatory cytokines and chemokines.

In addition, ECM proteins are overproduced, and ECM breakdown is inhibited during the development of fibrosis. ECM proteins are produced by various cell types in adipose tissue, including fat progenitor cells, adipocytes, fibroblasts, and myofibroblasts (144). Matrix metalloproteinases and tissue inhibitors of metalloproteinases govern ECM breakdown. Furthermore, matrix metalloproteinases can dissolve ECM components, which can be prevented by some tissue inhibitors of metalloproteinases (145).

Based on current research, necroptosis may be linked to adipose tissue fibrosis, and the essential molecular proteins of the necroptosis pathway could be key to controlling adipose tissue fibrosis. However, further experiments are required to verify this hypothesis.

In summary, our analysis of the relationship between fibrosis and necroptosis in wounds, liver, heart, lung, and adipose tissues illustrated that inhibiting necroptosis can significantly alleviate and improve the degree of fibrosis in tissues and organs. Therefore, in the next section, we review various ways to inhibit necroptosis.

## 3 Necroptosis inhibition in preventing tissue fibrosis

Considering the critical function of necroptosis in fibrosis control, pharmacological research on key molecules in the necroptosis pathway has been ongoing over the past decade, with promising findings. The first molecule shown to be a necroptosis regulator is Nec-1 (5, 146), which inhibits RIPK1 phosphorylation and indirectly inhibits RIPK1-RIPK3-MLKL signal transduction, preventing necroptosis and suppressing the inflammatory response. Nec-1 has displayed positive benefits in various disease models, including renal fibrosis (147), but its use is restricted due to its short *in vivo* half-life and nontargeting action (148). Nec-1 analogs have been discovered in recent years, with advantages and disadvantages identical to those of Nec-1 (147). Caspase inhibitors have been used with inhibitors of apoptosis antagonists (SMAC mimics) or TAK1 inhibitors (149, 150). Clinical studies of Nec-1 and its analogs are currently underway (151). GSK 840, GSK 842, GSK 872, GW 39B, and other inhibitors targeting RIPK3 have revealed promising results (152, 153). However, at high doses, several of these drugs cause RIPK3-dependent apoptosis (154), which limits their use to a certain extent. In addition, *in vitro* experiments have shown that necrosulfonamide can block necroptosis by inhibiting MLKL, another key regulatory factor in the necroptosis pathway (7). ICP6, M45, and immediate early gene 1, produced by some bacteria and viruses, can also affect the necrotic apoptosis pathway (155, 156). The above studies demonstrate that necroptosis and its regulatory mechanisms are highly valuable. However, more experiments and studies are still required before their application to clinical practice.

A similar effect can be achieved *via* DAMPs and their ligands, which suggests that they can improve the inflammatory response after tissue injury, promote tissue regeneration, and inhibit fibrosis formation by regulating necroptosis. Antibodies targeting DAMPs or their receptors can reduce alloimmunity, limit acute rejection, and limit the late development of chronic rejection-related graft vascular lesions and fibrosis (157). OPN-305 is a humanized IgG4 monoclonal antibody against TLR2 that prevents delayed graft function after renal transplantation. It also demonstrates a strong ability to antagonize TLR2 signal transduction activated by HMGB1 and heat shock proteins, which has been verified in animal models to inhibit ischemia-reperfusion injury (158, 159). Furthermore, OPN-305 can continuously inhibit the secretion of IL-6 by peripheral blood cells (160). The sialic-acid-binding immunoglobulin-like lectin-CD24 signaling pathway can inhibit DAMP-induced inflammation and weaken its proinflammatory function, particularly the secretion of TNF-α, IL-1b, and IL-6, to prevent the pathological inflammatory responses caused by cell death and necrosis (161, 162). Inhibition of HMGB1 can reduce fibroblast activation and destroy the process of fibrosis (84, 163). In addition, anti-HMGB1 antibodies can significantly reduce pulmonary fibrosis in animal models (72).

## 4 Necroptosis inhibition in stem cell therapy and engineered tissue construction

In recent years, an increasing number of studies have applied stem cell therapy for the treatment of fibrosis. Mesenchymal stem cells (MSCs) have been shown to reduce fibrosis in animal models of the lung (164), liver (165), kidney (166), heart (167), cavernous body, and urethra (168). However, stem cell therapy for fibrosis also has some defects; stem cells do not always exist in the recipient area, and the injected MSCs do not last long in the damaged tissue (169). Inflammatory and immune responses in the recipient area may induce MSC death (170). The local factors leading to stem cell apoptosis include membrane receptors, proteases, mitochondrial and nuclear proteins, growth factors, telomerase activity, and cell–cell signal transduction (171). Experiments have shown that MSCs are extremely sensitive to cell death induced by death-promoting molecules such as FasL (172). In addition to the mechanism of necroptosis mentioned above, stem cells are also associated with necroptosis, and the death and aging of stem cells are inhibited by inhibiting necroptosis to improve the effect of stem cell therapy and minimize fibrosis. Therefore, providing an appropriate microenvironment for stem cells is the key to ensuring the ideal outcome of stem cell therapy.

In addition to stem cell therapy, tissue engineering has rapidly developed in recent years. Organ transplantation is the only curative option for several fibrotic diseases. However, due to the shortage of donor organs and transplantation-related complications, the development of new treatments is necessary, and *in vitro* culture of engineered tissue has arisen as a potential alternative. However, fibrosis can still occur in engineered tissue. When engineered tissue is implanted into the body, the persistent chronic inflammation and immune response in the recipient area activate resident and recruited cells and induce the differentiation of stem cells into myofibroblasts, resulting in excessive deposition of ECM and persistent fibrosis (173, 174). This also limits the application of organizational engineering to a certain extent. Necroptosis plays an important role in regulating local or systemic inflammatory responses, especially persistent chronic inflammation, making it possible to achieve the desired effect in engineered tissue by regulating necroptosis.

## 5 Discussion

To date, conventional antifibrosis treatment has achieved very limited results; therefore, new treatments to prevent fibrosis are urgently needed. The recent discovery of necroptosis has provided a new method for fibrosis treatment. However, several problems remain to be solved. Compared to other types of programmed death, necroptosis demonstrates different characteristics in regulating inflammation. Although some experiments have shown that necroptosis is associated with human fibrotic diseases, there is still a lack of solid evidence to support this role. It is, therefore, necessary to identify more specific, sensitive, and reliable molecular markers of necroptosis. Crosstalk also exists between necroptosis and other cell death processes, such as apoptosis, ferroptosis, and pyroptosis (175). This suggests that different cell signaling pathways may be intrinsically involved with necroptosis signaling pathways. Therefore, it is necessary to clarify necroptosis regulatory networks in order to comprehensively understand the pathophysiological role of necroptosis in fibrotic diseases and ultimately enable the development of new therapeutic strategies.

Similarly, although DAMPs and their corresponding receptors can exacerbate inflammatory responses and fibrosis, not all DAMP levels are proinflammatory or profibrotic. Fibroblast-specific deficiency of TLR4 is protective against fibrosis; however, mice deficient in TLR4, TLR2, and TLR9 exhibited aggravated pulmonary fibrosis (176–178). Moreover, the same target can produce contradictory outcomes of pro-fibrosis or fibrosis inhibition, depending on the timing of the intervention. Unfortunately, the existing research has not addressed the issues mentioned above. Therefore, further research is required to uncover the relationship among necroptosis, DAMPs, and fibrosis and to provide a reference for later transformation into clinical treatment.

## Author contributions

XL and XC wrote the original manuscript. FL and XC provided the general idea and edited the manuscript. All authors contributed to the article and approved the submitted version.

## Funding

This work was supported by the National Natural Science Foundation of China (82072196 and 81871573), the China Postdoctoral Science Foundation–funded project (2020M672721), and the Science and Technology Program of Guangzhou, China (202201011571).

## Conflict of interest

The authors declare that the research was conducted in the absence of any commercial or financial relationships that could be construed as a potential conflict of interest.

## Publisher’s note

All claims expressed in this article are solely those of the authors and do not necessarily represent those of their affiliated organizations, or those of the publisher, the editors and the reviewers. Any product that may be evaluated in this article, or claim that may be made by its manufacturer, is not guaranteed or endorsed by the publisher.
