# Corrigendum: Examination of the role of necroptotic damage-associated molecular patterns in tissue fibrosis

**DOI:** 10.3389/fimmu.2022.1048026

**Published:** 2022-11-09

**Authors:** Xu Liu, Feng Lu, Xihang Chen

**Affiliations:** Department of Plastic and Cosmetic Surgery, Nanfang Hospital, Southern Medical University, Guangzhou, China

**Keywords:** necroptosis, RIPK3, inflammation, DAMPs, fibrosis

In the published article, there was an error in the Funding statement. The grant number for the Science and Technology Program of Guangzhou, China was displayed as “202201011577”. The correct grant number is “202201011571’’. The correct Funding statement appears below.

## Funding

This work was supported by the National Natural Science Foundation of China (82072196 and 81871573), the China Postdoctoral Science Foundation–funded project (2020M672721), and the Science and Technology Program of Guangzhou, China (202201011571).

The authors apologize for this error and state that this does not change the scientific conclusions of the article in any way. The original article has been updated.

## Publisher’s note

All claims expressed in this article are solely those of the authors and do not necessarily represent those of their affiliated organizations, or those of the publisher, the editors and the reviewers. Any product that may be evaluated in this article, or claim that may be made by its manufacturer, is not guaranteed or endorsed by the publisher.

